# SEASONAL VARIATION IN PEDIATRIC DERMATOSES

**DOI:** 10.4103/0019-5154.60351

**Published:** 2010

**Authors:** Sabyasachi Banerjee, Dwijendra Nath Gangopadhyay, Sukumar Jana, Mitra Chanda

**Affiliations:** *From the Department of Dermatology, Calcutta National Medical College, Kolkata, India.*

**Keywords:** *Age*, *season*, *pediatric dermatoses*

## Abstract

**Introduction:**

The under-five population is a unique and vulnerable component of our society that always demands special attention.

**Aims:**

Our present work aimed to study the seasonal variation, age-wise variation and distribution of lesions of common dermatoses of this age group.

**Materials and Methods:**

We clinically studied all fresh cases attending the skin OPD of our hospital for one month each from summer, rainy season and winter. Total number of patients was 879.

**Results:**

The top six skin diseases in our study were impetigo, miliaria, scabies, furunculosis, seborrheic dermatitis and papular urticaria. On statistical analysis, scabies and seborrheic dermatitis were more prevalent during winter while impetigo, furunculosis and miliaria were more during summer and rainy season. Papular urticaria was more frequent in the rainy season. Seborrheic dermatitis predominantly affected the infants while impetigo, furunculosis, miliaria and popular urticaria were commoner in older age groups.

**Conclusion:**

Distribution of lesions of common dermatoses will help diagnose difficult cases and extensive evaluation of the body parts which, by virtue of being commonly affected, are must-examine sites in under-five children.

## Introduction

A cursory glance at the age pyramid of our country with broad base and tapering top[1] reminds us of the significance of studying various aspects of diseases among infants and younger children. According to the National Family Health Survey (1998-99), under-five male and female population of India is 11.2% and 11.1% respectively.[2] The profile of their diseases varies widely among different geographical locations and over time. The patients attending the Dermatology outdoor of our hospital mostly are the underprivileged urban slum dwellers, more than 85% of whom are below poverty line. It's a common knowledge that type and amount of disease in any community are affected directly or indirectly by climate. Various climatic factors that may determine the incidence of skin diseases are cold, heat, light, sunshine and humidity.[3] Also, different degrees of exposure to external factors as well as different levels of functional development of skin may give rise to differential prevalence of dermatoses among infants, toddlers and children. In the present study, we tried to quantify and assess the significance of seasonal variation and trend of change with age of common dermatoses of under-five children attending our outdoor. We also evaluated the distribution of lesions on different body parts and tried to find out five most common sites of involvement of common childhood dermatoses.

## Materials and Methods

All the newly registered under-five patients of Dermatology OPD of Calcutta National Medical College, during three representative months, at peaks of our three main seasons, e.g., summer, rainy season and winter were included in the present study.

For each case, we filled up a pro-forma which included name, age, sex, socioeconomic status (below poverty line/average/good), detail history, general survey, details of cutaneous examination and provisional diagnosis. For examination purpose we divided the body into 12 parts -scalp, face, neck, chest, back, abdomen, buttocks, genitalia, upper limbs, hands, lower limbs and feet. Mucous membrane was not routinely examined. No routine laboratory investigation was undertaken.

During compilation of data we took up only the primary diseases. So, secondary bacterial infections or eczematization was not treated as a separate entity from the underlying primary conditions. Patients with multiple diseases were entered on more than one occasion.

Then we tabulated and analyzed the pooled data with the χ^2^ test.

## Results

The total number of patients under our study was 879, demographic profile of which is given in Table 1.

**Table 1:** Demographic profile of study patients

Age	Male	Female	Total
Infants (0 to 1 year)	95	98	193
Toddlers (1 to 3 years)	202	201	403
Preschool children (3 to 5 years)	142	141	283
	439	440	879

According to our study, the six most common skin diseases of under-five children are impetigo (221 cases), miliaria (190 cases), scabies (148 cases), furunculosis (133 cases), seborrheic dermatitis (118 cases) and papular urticaria (94 cases). These top six pediatric dermatoses are tabulated in Table 2, which shows the comparative prevalence of these diseases during summer, rainy season and winter.

**Table 2:** Seasonal variation in six common dermatoses

Name of disease	Summer (267 patients)		Rainy season (372 patients)		Winter (240 patients)	
			
	No. of cases	% of patients having the disease	No. of cases	% of patients having the disease	No. of cases	% of patients having the disease
Impetigo	86	32.21	112	30.11	23	9.58
Miliaria	91	34.08	99	26.61	0	0.00
Scabies	35	13.11	52	13.98	61	25.42
Furunculosis	58	21.72	71	19.09	4	1.67
Seborrheic dermatitis	17	6.37	33	8.87	68	28.33
Papular urticaria	18	6.74	62	16.67	14	5.83

On statistical analysis, prevalence of seborrheic dermatitis was significantly high during winter (*P* < 0.0001, χ^2^= 63.98). The same was true for scabies (*P* < 0.0001, χ^2^ = 17.44). On the other hand, furunculosis was significantly less prevalent during the winter (*P* < 0.0001, χ^2^ = 47.45), while there was not much difference between its prevalence in summer and rainy season (*P* = 0.4). Impetigo, too, was significantly less during winter (*P* < 0.0001, χ^2^ = 65.57), but almost same during summer and rainy seasons (*P* = 0.03).

Papular urticaria, however, was found to be significantly more common during rainy season when compared with winter (*P* < 0.0001 and χ^2^ = 15.74) as well as summer (*P* < 0.0001 and χ^2^ = 13.98). There was not much difference between its prevalence in summer and winter (*P* = 0.67). Miliaria was found to be a disease exclusively of summer and rainy seasons. But the prevalence did not significantly vary when those two seasons were compared (*P* = 0.04).

Table 3 shows the distribution of top six diseases among these age sub-groups of study patients.

**Table 3:** Age-wise breakup of common dermatoses

Diseases	Infants	Toddlers	Preschool children
Impetigo	25	120	76
Miliaria	28	108	54
Scabies	42	62	44
Furunculosis	13	73	47
Seborrheic dermatitis	60	41	17
Papular urticaria	9	52	33

On statistical analysis, seborrheic dermatitis significantly was more prevalent during infancy and decreased steadily with growing age (*P* < 0.0001 and χ^2^ = 68.87). Both furunculosis (*P* = 0.0008) and impetigo (*P* < 0.0001) occurred much less commonly in infants compared to other age groups. The same was true for papular urticaria (*P* = 0.0008). Scabies shows no significant trend with age difference. Miliaria, however, was found to be significantly more prevalent among toddlers than among infants (*P* < 0.0001) and preschool children (*P* = 0.02).

Less common dermatological conditions detected by us were (number of cases given bracket) as follows. Pediculosis capitis (35), candidiasis (23), pityriasis versicolor (19), dermatophytosis (8), insect bite (21), contact dermatitis (20), atopic dermatitis (16), pityriasis alba (13), nummular eczema (9), juvenile plantar dermatitis (4), asteatotic eczema (9), pompholyx (2), intertrigo (16), drug rash (11), urticaria (19), viral exanthem (2), molluscum contagiosum (15), viral wart (4), herpes labialis(4), Mongolian spots (32), congenital melanocytic nevus (19), nevus depigmentosus (10), hemangioma (4), epidermal nevus (1), lichen striatus (7), lichen spinulosus (7), lichen nitidus (3), lichen sclerosus *et.* atrophicus (3), lichen planus (2), vitiligo (9), icthyosis vulgaris (9), alopecia areata (3), scarring alopecia (2), erythema toxicum neonatorum (3), pityriasis rosea (3), palmoplanter keratoderma (3), psoriasis (2), acne (2), lupus vulgaris (2), scrofuloderma (1), acrodermatitis enteropathica (2), morphoea (1).

## Discussion

Majority of the general hospital's skin outpatient attendance in our country consists of infections which are acute and usually recurrent-scabies, pyodermas, pediculosis, parasitic and viral infections - and have been classified as diseases of poor economy.[4] The role of poverty, overcrowding, undernutrition, and consequent poor immunity are emphasized by these preventable diseases.

Figueroa *et l*. reported skin infestations as the most prevalent skin pathology in a study among school children in rural Ethiopia.[5] The high prevalence of pyogenic bacterial skin infection (40.27%) found in our study is quite similar to that reported by Ghosh SK *et al*. (35.6%).[6]

According to Dagan R, impetigo is the most common childhood skin infection.[7] The outcome of our study is also the same.

Hayden reported an overall prevalence of eczema as 20% with seborrheic dermatitis as 9% and contact dermatitis 5% in a hospital based study among pediatric population in the U.S.A.[8] In our study, overall prevalence of eczematous dermatoses was 22.98% whereas seborrheic dermatitis was the commonest form (13.42%). However, many times it becomes difficult to differentiate atopic dermatitis in infancy from infantile seborrheic dermatitis.[9]

Low temperature and low humidity in winter are among many of the presumed etiological factors of seborrheic dermatitis.[10] This may explain the high prevalence of the same during winter in our study. We found a significant clustering of cases of papular urticaria during the rainy season. The seasonal incidence may be attributed to the biting habits of the insects in the locality.[11] Rainy season is a favorable time for the breeding of insects. High temperature and humidity of summer and rainy seasons favors rapid proliferation of pyogenic bacteria, hence high prevalence of bacterial skin infections. According to Sahl and Mathewson, the incidence of impetigo is greatest during the summer when there is common close contact between children.[12]

Sebaceous secretion rates are high in neonates due to placental transfer of maternal androgens. Sebaceous gland activity decreases from the end of the first month.[13] It explains the relatively high incidence of seborrheic dermatitis in infancy and the steadily declining trend as the baby grows up.

We found the prevalence of impetigo, furunculosis, miliaria and papular urticaria shooting up suddenly when the patient enters the toddler age group. This may be attributed to the exposure to external environmental factors away from the cozy protection of home as well as increased physical contact with neighbors as the baby learns to walk. The finding also emphasized the high degree of parental care an infant usually commands. Later, diminution of prevalence of miliaria as the toddlers reach the pre-school-child age group may be due to development of some degree of tolerance to the environmental factors, the so called ‘hardening’ of the skin.

In our study, scabies most frequently affected the abdomen of males and upper limbs (especially front of wrist and antecubital fossa) of females. Scalp was the second most common site of involvement after face in case of impetigo as well as furunculosis. This high degree of localization of bacterial infection to scalp is presumably due to the habit of applying oil on scalp and hair which is rampant in the community under study.

We hope that the study of distribution of lesions of common dermatoses will help diagnose difficult cases and evaluate extensiveness of the disease by reminding us of the body parts which, by virtue of being commonly affected, are must-examine sites in under-five children.
